# Characterization of Human-Induced Neural Stem Cells and Derivatives following Transplantation into the Central Nervous System of a Nonhuman Primate and Rats

**DOI:** 10.1155/2022/1396735

**Published:** 2022-12-28

**Authors:** Mengjia Li, Zhengbo Wang, Tianqi Zheng, Tianzhuang Huang, Baoguo Liu, Deqiang Han, Sumei Liu, Bochao Liu, Mo Li, Wei Si, Y. Alex Zhang, Yuyu Niu, Zhiguo Chen

**Affiliations:** ^1^Cell Therapy Center, Beijing Institute of Geriatrics, Xuanwu Hospital Capital Medical University, National Clinical Research Center for Geriatric Diseases, And Key Laboratory of Neurodegenerative Diseases, Ministry of Education, Beijing, China; ^2^State Key Laboratory of Primate Biomedical Research, Institute of Primate Translational Medical, Kunming University of Science and Technology, Kunming, Yunnan 650500, China; ^3^College of Pharmacy, Jilin University, Changchun, Jilin 130021, China; ^4^Center of Parkinson's Disease, Beijing Institute for Brain Disorders, Beijing 100069, China; ^5^Faculty of Life Science and Technology, Kunming University of Science and Technology, Kunming, Yunnan 650500, China; ^6^Center of Neural Injury and Repair, Beijing Institute for Brain Disorders, Beijing 100069, China

## Abstract

Neural stem cells (NSCs) and derivatives are potential cellular sources to treat neurological diseases. In the current study, we reprogrammed human peripheral blood mononuclear cells into induced NSCs (iNSCs) and inserted GFP gene into the AAVS1 site for graft tracing. Targeted integration of GFP does not affect the proliferation and differentiation capacity of iNSCs. iNSC-GFP can be further differentiated into dopaminergic precursors (DAPs) and motor neuron precursors (MNPs), respectively. iNSCs were engrafted into the motor cortex and iNSC-DAPs into the striatum and substantia nigra (SN) of a nonhuman primate, respectively. The surviving iNSCs could respond to the microenvironment of the cortex and spontaneously differentiate into mature neurons that extended neurites. iNSC-DAPs survived well and matured into DA neurons following transplantation into the striatum and SN. iNSC-MNPs could also survive and turn into motor neurons after being engrafted into the spinal cord of rats. The results suggest that iNSCs and derivatives have a potential to be used for the treatment of neurological diseases.

## 1. Introduction

Neural stem cells (NSCs) [1] and their derivatives are promising cellular candidates for the treatment of various neurological diseases/disorders. The last decade has witnessed rapid progress in the reprogramming field that could provide alternative NSC sources in addition to fetal brain-derived primary NSCs [1–4]. In our previous study, we have reported the generation of iNSCs through reprogramming human peripheral blood mononuclear cells (PBMNCs) with Sendai viral vectors encoding key reprogramming factors and modifying the culture condition to NSC-selective medium [5]. The obtained iNSCs show a good capacity of self-renewal and differentiation to specific neuronal subtypes, such as dopaminergic (DA) neurons [6, 7].

Human iNSC- (hiNSC-) derived DA precursors exhibit safety and therapeutic effect when transplanted into an immunodeficient mouse Parkinson's disease (PD) model [7], suggesting a good potential for clinical application. Nevertheless, a test of hiNSC-DA precursors in a nonhuman primate model would be more informative. However, a good marker is lacking that can well differentiate human cells from monkey ones in a transplantation setting. Labelling of the graft with BrdU or nanoparticles has certain nonspecificity issues in that the labelled graft may transfer BrdU or nanoparticles to host cells once the graft dies [7–10]. Targeted knock-in of green fluorescent protein (GFP) may solve this problem.

Furthermore, it is less well-known whether iNSCs can be differentiated into other disease-relevant cell types. In the current study, we performed site-specific integration of GFP into iNSCs and characterized the iNSC-GFP and their derived DA and motor neuron (MN) precursors following engraftment into the central nervous system (CNS) of monkey and rat.

## 2. Materials and Methods

### 2.1. Isolation and Culture of PBMNCs

The isolation and expansion of PBMNCs has been previously reported [11]. ~5 ml of fresh blood was collected from the donor into an anticoagulant tube. The mononuclear cells were isolated by gradient centrifugation using Ficoll (Sigma-Aldrich, United States) and cultured in EM medium containing Stemspan 3000 (Invitrogen, United States), 50 *μ*g/ml ascorbic acid (Sigma-Aldrich, United States), 50 ng/ml SCF (Miltenyi, Germany), 10 ng/ml IL-3 (Invitrogen, United States), 2 U/ml EPO (R&D Systems, United States), 40 ng/ml IGF-1 (Miltenyi, Germany), and 1 *μ*m dexamethasone (Sigma-Aldrich, United States).

### 2.2. Reprogramming of PBMNCs to iNSCs

To the reprogramming of PBMNCs to iNSCs, 5 × 10^5^ PBMNCs were mixed with 20 *μ*l Sendai reprogramming vectors encoding Oct3/4, Sox2, Klf4, and Myc (Thermo Fisher Scientific, United States). The next day, the Sendai reprogramming vectors were removed by changing the medium. On the third day, the medium was changed to NSC medium consisting of DMEM/F12, Neurobasal in 1 : 1 ratio, 1 × N2, 1 × B27, 1% GlutaMAX™, 1% penicillin/streptomycin, 1% NEAA (all from Thermo Fisher Scientific, United States), human recombinant leukemia inhibitor factor (hrLIF, Millipore, MA, United States), and SB431542 and CHIR99021 (both from Selleck, United States). On the 7th day or so, the iNSC clones would appear. Three of the emerging clones were manually picked for further passaging. The three clones were characterized for their growth curves, NSC marker expression, and differentiation capacity, and all three showed comparable phenotypes (data not shown). Therefore, one of the three clones was randomly selected for GFP knock-in and subsequent transplantation experiments.

### 2.3. Targeted Integration of GFP at the AAVS1 Site

The iNSCs were dissociated into single cells by using Accutase. Following the manufacturer's instructions of the 4D-Nucleofector system (Lonza, Switzerland), the Cas9-expressing plasmids and donor plasmids containing the homologous arms as well as GFP-coding sequence were placed into the 4D-Nucleofector system together with the electrotransfer buffer (the Cas9-expressing plasmid was constructed by the authors, and the donor plasmids were purchased from Addgene, United States). 48 hours following electroporation, the expression of green fluorescence was observed. The cells with green fluorescence were sorted using flow cytometry. Homozygous GFP-expressing cells were further selected by single-cell culture and clone expansion.

### 2.4. Differentiation of iNSCs to Dopaminergic Neurons

iNSCs were dissociated into single cells by using Accutase and placed into PDL/laminin-coated plates. A two-step differentiation method was used to differentiate iNSCs into DA neurons. During the first stage, iNSCs were treated with SAG1 (Enzo, United States) and fibroblast growth factor 8 (FGF8, PeproTech, United States) for 10 days. During the second stage, the medium was replaced with a mixture containing ascorbic acid (AA, Sigma-Aldrich, United States), brain-derived neurotrophic factor (BDNF, PeproTech, United States), glial cell line-derived neurotrophic factor (GDNF, PeproTech, United States), cyclic adenosine monophosphate (cAMP, Sigma-Aldrich, United States), transforming growth factor *β*III (TGF-*β*III, PeproTech, United States), and DAPT (Sigma-Aldrich, United States) for another 14 days to facilitate the maturation of DA neurons.

### 2.5. Differentiation of iNSCs to Motor Neurons

iNSCs were dissociated into single cells and placed into Matrigel (Corning, United States)-coated plates. The differentiation process was divided into three stages. During the first stage, the iNSC medium was replaced with a chemically defined neural medium, including DMEM/F12, Neurobasal medium in 1 : 1 ratio, 0.5 × N2, 0.5 × B27, 0.1 mM ascorbic acid (Sigma-Aldrich, United States), 1% GlutaMAX™, and 1% penicillin/streptomycin. 1 *μ*M CHIR99021, 2 *μ*M DMH-1 (Tocris), and 2 *μ*M SB431542 were added in the medium. Simultaneously, retinoic acid (RA, 0.1 *μ*mol/l, Stemgent, United States) and inhibitor of SHH pathway (purmorphamine, 0.5 *μ*mol/l, Stemgent, United States) were added to the medium for 7 days. The culture medium was changed every other day. iNSCs maintained under this condition for 6 days and differentiated into OLIG2-positive motor neuron precursor cells. In the next stage of differentiation, we withdrew CHIR+SB+DMH, increased RA concentration to 0.5 *μ*mol/l, and reduced purmorphamine to 0.1 *μ*mol/l. The medium was changed every other day. OLIG2-positive MNPs under this condition for 6 days differentiated into HB9-positive MNs. In the last stage of differentiation, the cultures were digested by Accumax (Invitrogen, United States) on laminin-coated plates and treated with compound E (Selleck, United States), which is a NOTCH signaling inhibitor. HB9-positive MNs gradually mature and interweave into a network structure.

### 2.6. Immunofluorescence Staining of Cultured Cells

Cells were washed 3 times with PBS and fixed by using 4% paraformaldehyde for 15 min. The cells were then blocked with a mixture of 3% fetal bovine serum and 0.05% Triton X-100 for 1 hour. The cells were incubated with the primary antibodies overnight at 4°C followed by 3 washes. Then, the secondary antibodies were added followed by nuclear counterstaining with DAPI for 2 hrs at room temperature. The stained cells were imaged by using a SP8 Leica confocal microscope. The antibody information is listed in Supplementary Table S2.

### 2.7. Karyotype Analysis

Karyotype analysis was carried out as described before [12, 13]. Briefly, iNSCs were cultured in NSC medium for 2 days, and then, the cells were incubated with 100 ng/ml colcemid (Life Technologies, United States) for 90 min. Then, the cells were fixed with an ice-cold methanol-glacial acetic acid (3 : 1) solution for 3 times. Chromosomes were stained with Hoechst (Life Technologies, United States) and counted under 630x magnification.

### 2.8. Animal Surgery and Cell Transplantation

For the nonhuman primate experiment, one healthy female rhesus monkey was used in the current study. The animal was anesthetized by intramuscular injection of ketamine hydrochloride (5-10 mg/kg of animal body weight) in combination with dormium (1 mg/ml). To suppress the inflammatory response, daily intramuscular injection of penicillin (800, 000 U/day) was required preoperatively and continued for additional three days postoperatively. During surgery, a 5% glucose solution was supplied and titrated to maintain the animal's energy and fluid balance. An injection of Tolfedine CS (0.1 ml/kg) was performed to relieve the animal's pain.

During surgery, the head of the macaque was fixed by using a stereotaxic instrument. 3 × 10^6^ DA precursors suspended in 30 *μ*l DPBS with 100 ng/ml BDNF, 100 ng/ml GDNF, and 4 ng/ml bFGF were injected into the right-hand side striatum (position of the first point: A/P + 28.4 mm, M/L + 4.8 mm, and D/V-11.4 mm; position of the second point: A/P + 23.0 mm, M/L + 5.1 mm, and D/V-11.3 mm) and substantia nigra (A/P + 10.8 mm, M/L + 4.8 mm, and D/V-33 mm), respectively, as guided by using MRI images. In the meantime, 3 × 10^6^ iNSCs suspended in 30 *μ*l buffer as mentioned above were injected into the motor cortex area of the right prefrontal lobe (A/P + 38.0 mm, M/L + 3.0 mm, and D/V-1.2 mm).

iNSC-derived motor neuron precursors were transplanted into the T9-T10 region of the anterior horn gray matter of three rats, with 8 × 10^5^ MN precursors suspended in 30 *μ*l DPBS injected into each spinal cord. Surgery was performed by anesthetizing the animals with 1% sodium pentobarbital at an i.p. dose of 30 mg per kg body weight.

### 2.9. Immunosuppressant Injections

The transplanted animals were injected with the immunosuppressant cyclosporine A (Novartis, Switzerland) three days before the cells were transplanted. Cyclosporine A was injected twice daily at a dose of 10 mg/kg/day. Animals receiving cell transplants were treated with cyclosporine A to suppress immune rejection until they were euthanized.

### 2.10. Frozen Sectioning of Tissues and Immunofluorescence Staining

Three months following transplantation of cells into different macaque brain regions, the animal was euthanized. The brain was removed after perfusion and fixed with 4% paraformaldehyde, followed by dehydration with 30% sucrose. The tissues were embedded with O.C.T. compound (Sakura Finetek, Japan) for at least 30 min in a -20°C freezer and then sectioned in the sagittal plane using a freezing microtome. The tissue sections were immunofluorescence stained according to the method described above. The antibody information is listed in Supplementary Table S2.

Similarly, two weeks after transplantation, rats were euthanized, and spinal cord tissues were fixed by perfusion, sectioned, and then immunofluorescence stained as described above.

### 2.11. Statistical Analysis

Statistical analysis and graphing were performed by using GraphPad Prism 8.0.2 (GraphPad Software, La Jolla, CA). The data were presented as mean ± SEM. One-way ANOVA was used to compare three or more groups, followed by Dunnett's multiple comparison test. And a *p* value ≤ 0.05 was considered as significant.

To quantify the proportion of cells positive for specific markers, three cultures of cells derived from the same clone were subjected to immunostaining and quantification, in which 9 fields of each culture were sampled and analyzed using the ImageJ software (U.S., NIH).

To quantify the surviving grafted cells in animals, one out of every 5 consecutive sections (20 *μ*m thick) across the entire graft site was counted.

## 3. Results

### 3.1. PBMNCs Are Reprogrammed into iNSCs by Using Sendai Vectors

**Figure 1:**
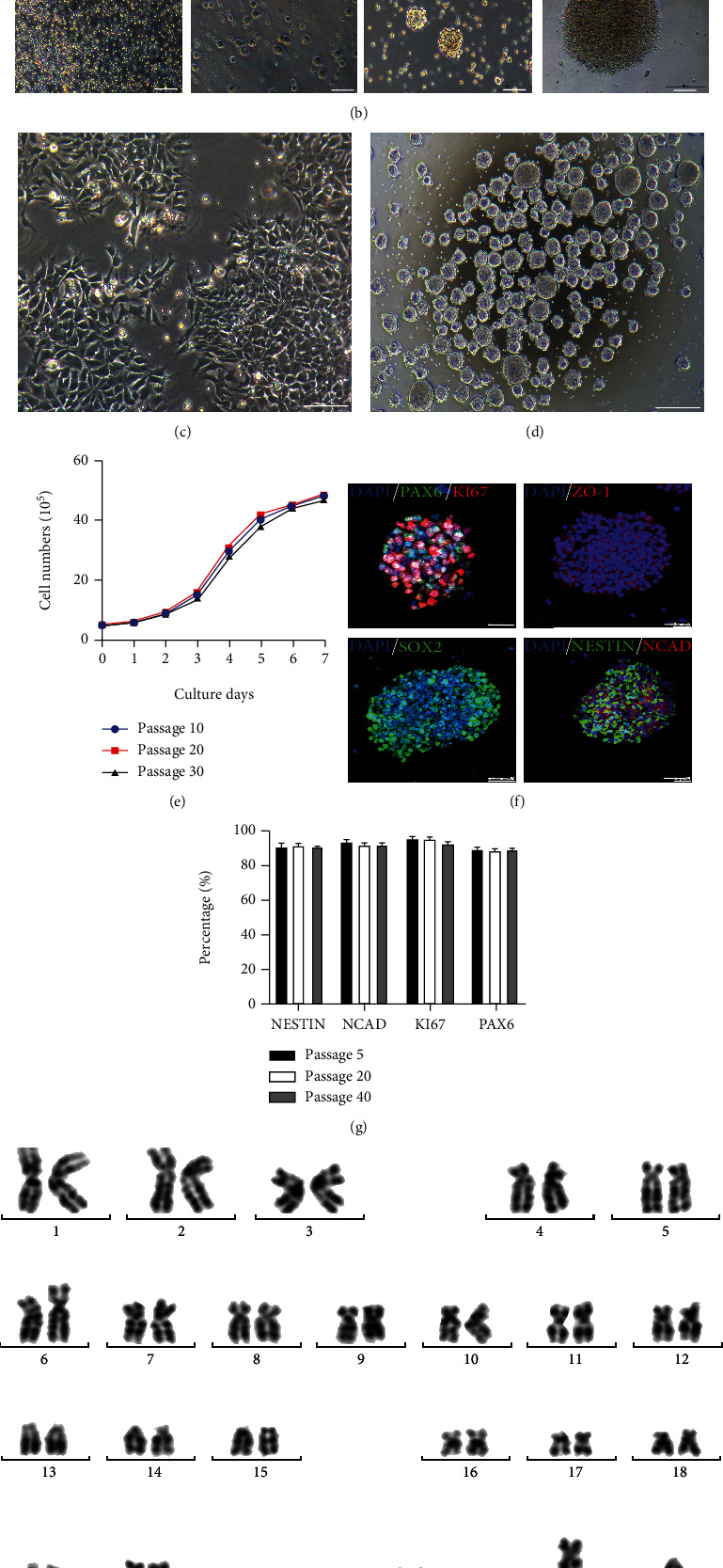
PBMNCs are reprogrammed into iNSCs. (a) The flowchart is about the procedure converting PBMNCs to iNSCs by using Sendai reprograming vectors expressing the Yamanaka factors. (b) Representative pictures of cells at different time points. Scale bars, 200 *μ*m. (c) A representative picture of the induced iNSCs cultured as a monolayer. Scale bars, 200 *μ*m. (d) A representative picture of the induced iNSCs cultured as spheres. Scale bars, 500 *μ*m. (e) One-week expansion curves of iNSCs of passage nos. 10, 20, and 30. (f) iNSCs stained positive for markers SOX2, N-CADHERIN (NCAD), PAX6, NESTIN, ZO-1, and KI67. Scale bars, 50 *μ*m. (g) Percentages of iNSCs at passage nos. 5, 20, and 40 that were positive for NESTIN, NCAD, KI67, and PAX6, respectively. (h) Karyotype analysis of iNSCs.

Peripheral blood mononuclear cells (PBMNCs) were isolated from peripheral venous blood (5 ml) of an adult healthy male donor through gradient centrifugation as previously reported [11]. After culture for 5 days, the PBMNCs were transduced with Sendai reprogramming vectors encoding Oct3/4, Sox2, Klf4, and Myc [14]. The cells were then transferred onto PDL/laminin-coated plates in a specific medium containing human recombinant leukemia inhibitor factor (hrLIF), SB431542, and CHIR99021 (Figure 1(a)). On the seventh day posttransduction, NSC-like clones started to appear and were manually picked for expansion *in vitro*. The morphology of cells at different time points posttransduction is shown in Figure 1(b). These iNSCs could steadily self-renew in a monolayer adherent form on PDL/laminin-coated plates as well as in a neurosphere form on a low adhesion culture plate (Figures 1(c) and 1(d)). The iNSCs still maintained a comparable growth rate after 30 passages (Figure 1(e)). In the following, we determined whether the transdifferentiated cell line expressed the signature proteins of neural stem cells. By using immunofluorescence staining, iNSCs were confirmed to be positive for PAX6, NESTIN, ZO-1, SOX2, N-CADHERIN (NCAD), and KI67 (Figure 1(f)). The proportion of cells positive for KI67, NESTIN, PAX6, and NCAD reached about 90%, and there was no significant difference between cells of passage number 5, 20, and 40 (Figure 1(g)). In addition, the obtained iNSCs showed a normal karyotype (Figure 1(h)).

### 3.2. Crispr-Cas9-Mediated Targeted Integration of GFP at the AAVS1 Site

**Figure 2:**
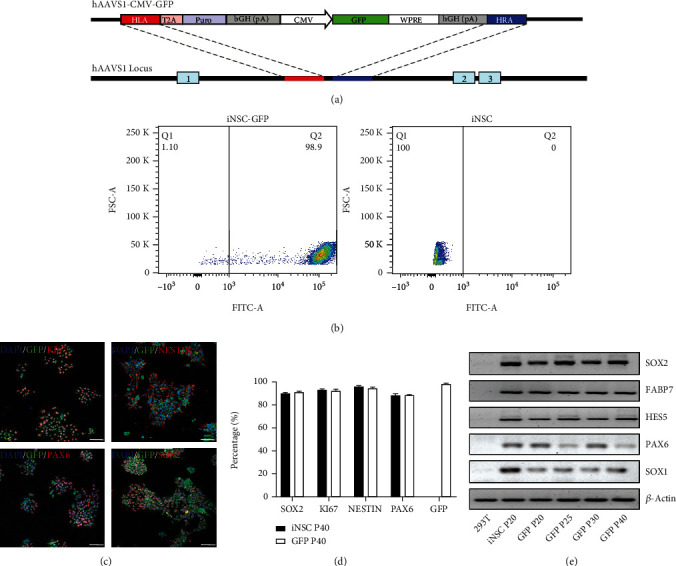
Crispr-Cas9-mediated targeted integration of GFP at the AAVS1 site. (a) Schematic diagram of the knock-in strategy showing the GFP-containing homologous recombination sequence (hAAVS1-CMV-GFP) and the insertion site (hAAVS1 locus). (b) Flow cytometric analysis results of iNSC-GFP and control iNSCs without GFP knock-in. (c) Immunofluorescence staining for GFP, NESTIN, KI67, PAX6, and SOX2. Scale bars, 75 *μ*m. (d) The percentages of iNSCs at passage no. 40 with or without GFP knock-in that were positive for SOX2, KI67, NESTIN, PAX6, and GFP. (e) Expression of SOX1, SOX2, PAX6, HES5, FABP7, and *β*-actin of 293T, iNSCs of passage no. 20, and iNSC-GFP of passage nos. 20, 25, 30, and 40 at the transcription level.

GFP sequence was inserted into the AAVS1 locus by using a homologous recombination approach mediated by Crispr-Cas9 system [15]. Briefly, Cas9-expressing plasmid and a donor plasmid containing homologous arms and GFP-coding sequence were introduced into iNSCs by electron transfection (Figure 2(a)). iNSCs with GFP integration were confirmed by PCR using three specific pairs of primers (Figure S1A-B). Expression of GFP could be detected in iNSC-GFP cultured in a proliferative medium or differentiation medium (Figure S1C). By flow cytometric analysis, GFP-positive cells reached 98.9%, and GFP expression was negative in the control group of iNSCs without GFP knock-in (Figure 2(b)). By using immunofluorescence staining, iNSC-GFP remained positive for SOX2, PAX6, NESTIN, and KI67 (Figure 2(c)). In addition, iNSC-GFP was negative for pluripotency markers NANOG and SSEA4 (Figure S1D). NSC markers were also confirmed to be expressed by iNSC-GFP at the transcription level by quantitative reverse transcriptase polymerase chain reaction (qRT-PCR) technique (Figure 2(e)). The results showed that iNSC-GFP could stably express GFP without affecting the characteristics of neural stem cells.

### 3.3. iNSC-GFP Can Differentiate into Midbrain Dopamine Neurons with High Efficiency *In Vitro*

**Figure 3:**
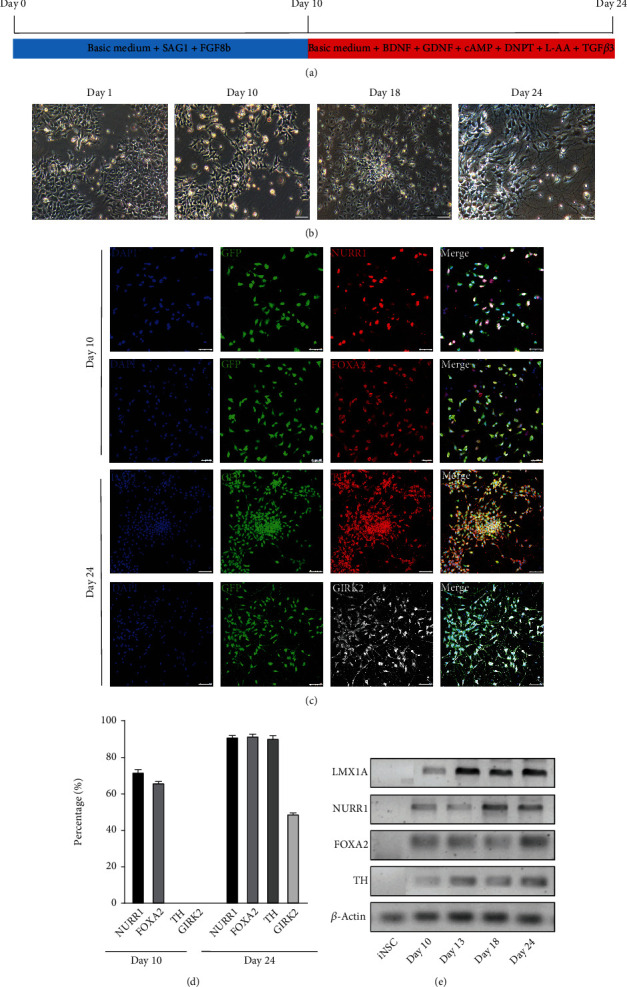
Differentiation of iNSCs into midbrain dopamine neurons with high efficiency in vitro. (a) Flow chart of the differentiation of iNSCs into DA neurons in vitro. (b) Representative pictures of cells at different differentiation time points. (c) Immunofluorescence staining for NURR1 and FOXA2 on cells of differentiation day 10 (scale bars, 50 *μ*m) and for TH and GIRK2 on cells of differentiation day 24 (scale bars, 75 *μ*m). (d) Quantification of positive cells for each marker. (e) Expression of LMX1A, NURR1, FOXA2, TH, and *β*-actin at the transcription level in iNSC-GFP before differentiation and at differentiation days 10, 13, 18, and 24.

To examine the differentiation capacity of iNSC-GFP, we employed a protocol to differentiate iNSCs to DA neurons (Figure 3(a)) [7]. The morphology of cells at different time points is shown in Figure 3(b). DA neural lineage-related markers were examined on cells of differentiation day 10 and day 24 (Figure 3(c)). On differentiation day 10, cells barely expressed tyrosine hydroxylase (TH) or G-protein-regulated inward-rectifier potassium channel 2 (GIRK2), both of which are markers of mature midbrain dopamine neurons [16]. At this time point (day 10), a majority of cells were positive for the relatively earlier DA neural markers, fork head box protein A2 (FOXA2, 66.33%) and nuclear receptor related-1 protein (NURR1, 71.5%) (Figures 3(c) and 3(d)). On differentiation day 24, the proportions of FOXA2- and NURR1-positive cells increased to 92.1% and 91.5%, respectively (Figures 3(c) and 3(d)). Meanwhile, the proportion of TH-positive cells reached 90.8%, and the proportion of GIRK2-positive cells increased to 32.63% (Figure 3(d)). Additionally, the transcription levels of DA lineage-related genes were measured by qRT-PCR on cells of differentiation days 10, 13, 18, and 24 (Figure 3(e)). The results showed that iNSCs can differentiate into dopaminergic neurons *in vitro* with high efficiency.

### 3.4. iNSC-GFP Can Be Differentiated into Motor Neurons *In Vitro* with High Efficiency

**Figure 4:**
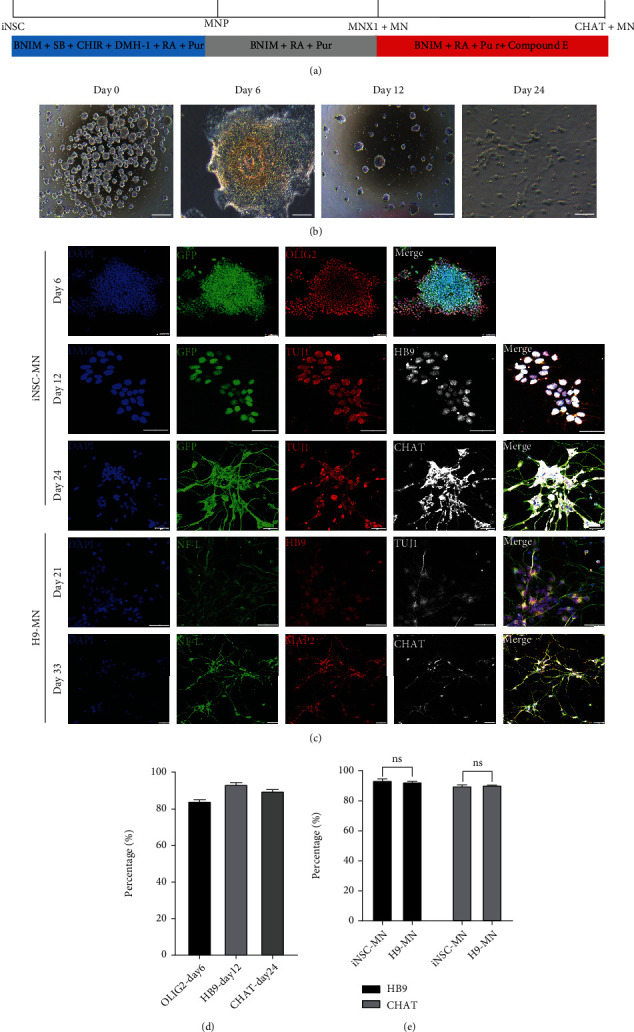
Differentiation of iNSCs into motor neurons in vitro. (a) Flow chart of the differentiation of iNSCs into motor neurons in vitro. (b) Representative pictures of cells at different differentiation time points. (c) Immunofluorescence for OLIG2, HB9, and CHAT on iNSC-GFP-differentiated and embryonic stem cell line H9-differentiated cells at different time points, respectively. Scale bars, 50 *μ*m. (d) Quantification of differentiated iNSCs positive for OLIG2, HB9, and CHAT at different differentiation time points. (e) Comparison of the efficiencies of motor neuron differentiation from iNSC-GFP versus ESCs (H9 line).

We also tested whether iNSC-GFP can be differentiated into motor neurons. The differentiation process was divided into three stages as shown in Figure 4(a), and the representative pictures at different differentiation time points are shown in Figure 4(b). Next, we compared the differentiation into motor neurons; that is, the cells at each stage were identified by immunofluorescence. On differentiation day 6, more than 80% of the cells in the iNSC-GFP group were positive for the MN progenitor cell marker Olig2 (Figures 4(c) and 4(d)), and the percentages of HB9-positive and CHAT-positive cells peaked on differentiation day 12 (93.78%) and day 24 (90.12%), respectively (Figures 4(c) and 4(d)). As to the motor neuron differentiation from the embryonic stem cell line H9, the proportions of HB9-positive and CHAT-positive cells peaked at differentiation days 21 and 33, respectively (Figures 4(c) and 4(e)). There was no significant difference as to the peak proportions of HB9-positive and CHAT-positive cells differentiated from iNSC-GFP vs. H9 (Figure 4(e)). The results showed that iNSC-GFP can be efficiently and rapidly differentiated into MN *in vitro*.

### 3.5. Characterization of Grafted iNSCs and iNSC-DAPs Three Months following Transplantation into Different Areas of the Nonhuman Primate

**Figure 5:**
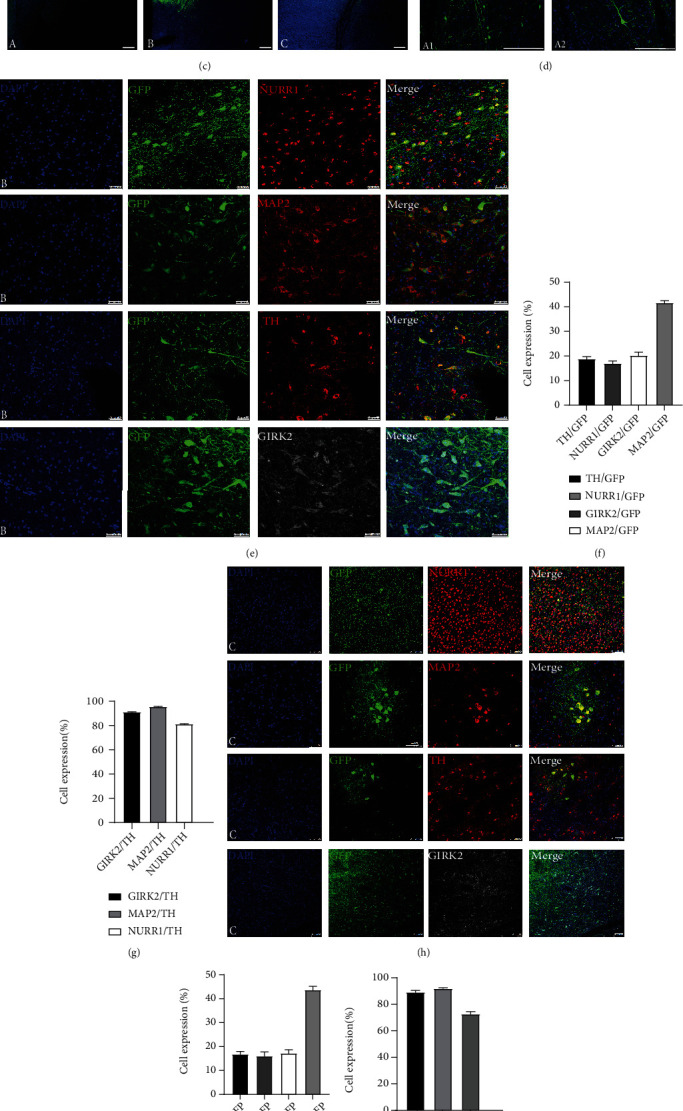
Characterization of cells following engraftment into different brain regions of a nonhuman primate. (a) The schematic diagram of the transplantation experiment. (b) The three brain regions selected for transplantation with region a (motor cortex) engrafted with iNSC-GFP, region b (striatum) engrafted with iNSC-DAPs, and region c (substantia nigra) engrafted with iNSC-DAPs. (c) The GFP-positive cellular grafts were detected at the three engraftment sites three months following transplantation. Scale bars, 1 mm. (d) Three months following transplantation, the engrafted iNSC-GFP at the motor cortex showed the morphology of neurons. Scale bars, 250 *μ*m. (e) Immunofluorescence staining for NURR1, MAP2, TH, and GIRK2 on the transplanted iNSC-DAPs three months following engraftment into the striatum. Scale bars, 50 *μ*m. (f) Three months after transplantation of iNSC-DAPs, quantification of the GFP-positive cells that coexpressed TH, NURR1, GIRK2, and MAP2, respectively. (g) The proportions of TH-positive cells that coexpressed GIRK2, MAP2, and NURR1 three months following transplantation of iNSC-DAPs into the striatum. (h) Immunofluorescence staining for NURR1, MAP2, TH, and GIRK2 three months following engraftment of iNSC-DAPs into the substantia nigra. Scale bars, 100 *μ*m. (i) The proportions of surviving cells that were double-positive for GFP and TH, NURR1, GIRK2, or MAP2, respectively, at the substantia nigra. (j) The proportions of TH-positive cells that coexpressed GIRK2, MAP2, or NURR1 at the substantia nigra.

Next, we transplanted iNSCs and iNSC-DAPs into different brain regions of a nonhuman primate (Figure 5(a)). iNSCs were engrafted into the motor cortex and iNSC-DAPs to the striatum and substantia nigra (SN) (Figure 5(b)). Three months following transplantation, the brain tissue was sliced and analyzed by immunofluorescence staining. GFP-positive grafted cells were detected at the injection sites (Figure 5(c) A, cortex; Figure 5(c) B, striatum; and Figure 5(c) C, SN). In the cortex, GFP-positive cells with neuronal morphology were observed. The neurons showed long processes (Figure 5(d)), suggesting that iNSCs could react to the cortex niche and differentiate to mature neurons. In Parkinson's disease, dopaminergic neuronal circuits that control motor functions were damaged, and these DA neurons and projection targets are located in the SN and striatum. Given the relevance to PD, iNSC-DAPs were engrafted into the striatum and SN. Three months following engraftment, GFP-positive cells in the striatum matured into DA neurons that were positive for TH, GIRK2, NURR1, and the universal neuronal marker microtubule-associated protein 2 (MAP2) (Figure 5(e)). In the striatum, around 18.82%, 17.03%, 20.25%, and 41.70% of the GFP-positive cells coexpressed TH, NURR1, GIRK2, and MAP2, respectively (Figure 5(f)). About 91.25%, 95.57%, and 81.32% of the TH-positive cells were colabelled with GIRK2, MAP2, and NURR1, respectively (Figure 5(g)). Similarly, iNSC-DAPs transplanted into the SN were analyzed by using immunofluorescence staining (Figure 5(h)). Among the GFP-positive grafted cells, about 16.82%, 17.22%, 16.03%, and 45.35% coexpressed TH, GIRK2, NURR1, and MAP2, respectively. Among the TH-positive grafted cells, about 89.30%, 92.0%, and 72.80% were colabelled with GIRK2, MAP2, and NURR1, respectively (Figures 5(i) and 5(j)). GIRK2 is a SN A9 region-specific DA neuronal marker, and the results suggested that the engrafted iNSC-DAPs had matured into midbrain A9 DA neurons following transplantation into the striatum and SN of the brain of a nonhuman primate.

To examine the safety and lineage specificity of the engrafted cells, we analyzed the expression of KI67, SOX2, and NESTIN in the motor cortex and the expression of VGluT1, a glutaminergic neuronal marker, CHAT, a cholinergic neuronal marker, and GFAP, an astrocyte marker, in the striatum and SN (Figure S2). In the motor cortex, no GFP-positive cells were found to be colabelled with KI67, SOX2, or NESTIN, suggesting that the grafted iNSCs had exited the cell cycle and lost stemness three months following transplantation—a sign of no tumorigenicity. A few SOX2-positive cells were detected, but those cells were negative for GFP. We also investigated whether iNSC-DAPs transplanted into the striatum and SN might have turned into other neural lineages. However, no GFP-positive cells were detected to coexpress VGluT1, CHAT, or GFAP.

**Figure 6:**
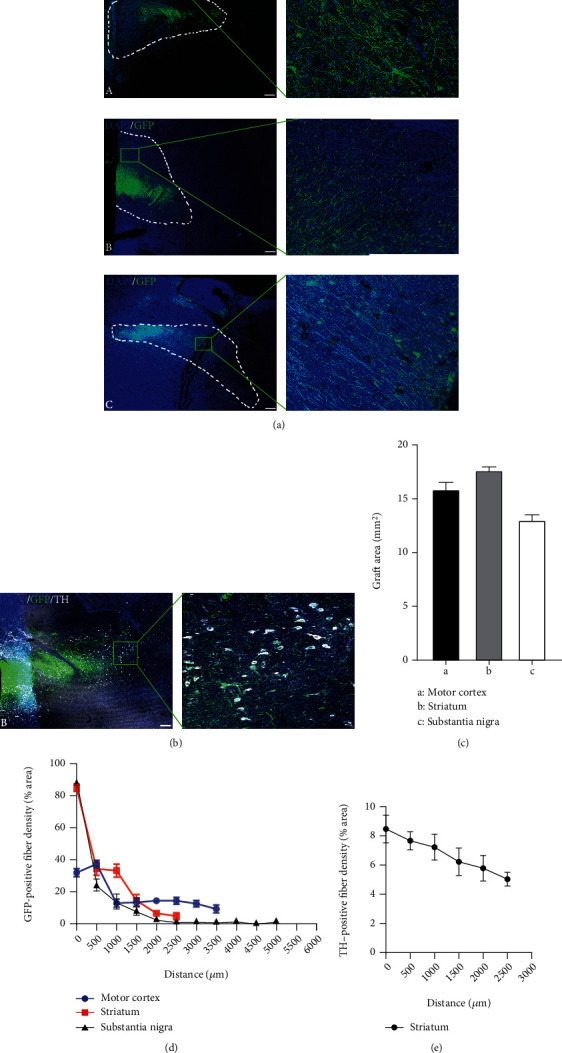
Neurite/axon projection of grafted cells at different brain regions three months following transplantation. (a) GFP-positive cellular grafts were detected in the motor cortex (A), striatum (B), and substantia nigra (C), as well as the magnified insets at areas away from the graft bolus. Scale bars, 1 mm. (b) Immunofluorescence staining of TH. The inset showed the GFP-positive cells that were also positive for TH. Scale bars, 500 *μ*m. (c) The dotted line represents the area of the graft were measured 3 months following transplantation into the brain of the nonhuman primate. (d) Quantification of GFP-positive neurite density as to the distance to the graft injection sites at the three brain regions. (e) Quantification of GFP/TH double-positive neurite density as to the distance to the graft injection site at the striatum.

Next, we analyzed the neurite extension/projection of the grafted cells. Neurite extension was observed in three regions of the nonhuman primate brain three months following transplantation (Figure 6(a)). In the striatum, GFP/TH double-positive DA neurons also showed a morphology with neurite extension. Based on the spread of GFP-positive cells in brain tissue slices, we measured the areas of GFP-positive grafts in these three brain regions, and the graft areas at the motor cortex, striatum, and substantia nigra were 15.89 mm^2^, 17.67 mm^2^, and 13.02 mm^2^, respectively (Figure 6(c)). The neurite density of GFP-positive cells was also plotted against the distance to the graft bolus center. The neurite density decreased as the distance increased from the graft center, and neurites could be detected up to 5000 *μ*m away from the center of the SN and up to 2500 *μ*m at the striatum (Figure 6(d)). Neurite density was also measured on the GFP/TH double-positive cells in the striatum (Figure 6(e)).

### 3.6. Survival and Differentiation of MN Progenitors Transplanted into the Spinal Cord of Rats

**Figure 7:**
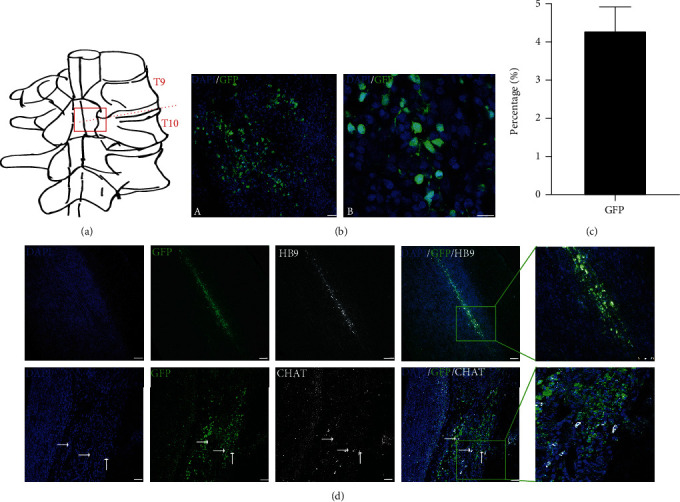
Survival and differentiation of iNSC-derived motor neuron progenitors transplanted into the spinal cord of rats. (a) Schematic diagram of transplantation sites in rat spinal cord. (b) The GFP-positive surviving grafts two weeks following transplantation into the spinal cord. Scale bars, 100 *μ*m. (c) The proportion of GFP-positive surviving cells. (d) Two weeks after transplantation, GFP-positive grafts were detected at the spinal cord that were also HB9- or CHAT-positive. Scale bars, 100 *μ*m.

Motor neuron progenitors are a potential candidate for the treatment of spinal cord disorders, such as ALS and spinal cord injury. We transplanted human iNSC-derived motor neuron progenitors into the spinal cord (T9-T10) of rats to characterize the survival and differentiation of iNSC-MNPs (Figure 7(a)). The rats received an immunosuppression regime for 3 days prior to transplantation daily until the end of the experiment. We first searched for the optimal differentiation time points that could give rise to a good graft survival. iNSCs were subjected to MN differentiation for 10, 12, and 14 days, and those cells of different differentiation time points were transplanted into the spinal cord of immune-suppressed rats for 2 weeks to test the graft survival. MNPs differentiated for 12 days gave rise to the best survival (data not shown). Two weeks after cell transplantation, which is day 12 of cell differentiation, about 4% of grafted cells survived in the spinal cord (Figures 7(b) and 7(c)). At this time, some grafted GFP-positive cells stained positive for the motor neuron markers, HB9, and CHAT (Figure 7(d)). The results indicated that iNSC-derived motor neuron progenitors could survive and differentiate into motor neurons after transplantation into the rat spinal cord.

## 4. Discussion

The induced neural stem cells (iNSCs) used in the current study were reprogrammed from human PBMNCs, a starter cellular source that is easily accessible in regular clinical practice. PBMNC-derived iNSCs possess a good potential to treat neurological diseases and meanwhile pose lower risk of tumorigenicity vs. pluripotent stem cells, such as embryonic stem cells (ESCs) and induced pluripotent stem cells (iPSCs). ESCs and iPSCs possess a good proliferative capacity and can be expanded almost infinitely *in vitro*, rendering them a good candidate of allogeneic “off-the-shelf” cellular drug. Indeed, in 2021, BlueRock Therapeutics has started a clinical trial and, for the first time, transplanted ESC-derived DAP (MSK-DA01, ClinicalTrials.gov Identifier: NCT04802733) into a PD patient [17]. The trial has brought hope and promise in many PD patients. Yet years of follow-up is required to examine the long-term safety and efficacy of the graft, particularly the potential tumorigenic risk associated with the possible incomplete differentiation of pluripotent stem cells. In addition, the impact of immune recognition of the allogeneic ESC-derived DAPs needs to be scrutinized. Previous clinical studies using fetal ventral mesencephalon tissues, as pioneered by Lindvall et al. and Lindvall and Kokaia [18, 19], have shown that allograft-induced immune response may affect the optimal graft functionality and may even be associated with some side effects [20–24]. In this regard, iPSCs are advantageous since they can be employed as autologous grafts [25–27]. Schweitzer et al. have tested iPSC-derived autologous DAPs in a PD patient, and the symptoms stabilized or improved at 18 to 24 months after implantation [28]. Compared with pluripotent stem cells (ESCs and iPSCs), iNSCs retain an intrinsic property similar to that of tissue stem cells and are thus considered to pose less tumorigenic risk. Indeed, direct transplantation of iNSCs into the brain of immunodeficient mice fails to result in tumor formation [7]. iNSCs can be used as autologous grafts as iPSCs do and normally require a relatively shorter period of time for reprogramming and specific differentiation [7]. iNSCs can also avoid the ethical, logistical, and heterogeneity issues associated with fetal brain tissues [29–33].

Before iNSCs and derivatives can be tested in clinical trials, transplantation studies employing nonhuman primates would be mostly informative as to reveal the safety and efficacy of these cells [34]. Nevertheless, a good marker is lacking that can well differentiate human vs. monkey cells. To address this issue, we inserted GFP at the specific genomic locus—AAVS1. Knock-in of GFP at AAVS1 did not affect the basic features of iNSCs, such as the proliferative and differentiation capacity. Upon transplantation, the grafted cells could be easily distinguished by the green fluorescence. Despite being a xenograft, human iNSCs showed robust survival following transplantation into the motor cortex of a monkey. No tumor formation was observed three months following transplantation of iNSCs, suggesting a good clinical potential. Interestingly, iNSCs seemed to be able to react to the local adult brain environment and differentiate into neurons with a morphology similar to a pyramidal neuron. It would be interesting to investigate whether iNSCs could automatically react to different disease conditions, such as brain trauma, stroke, and epilepsy, and differentiate into relevant functional cells accordingly; future studies are warranted to answer this question.

iNSC-derived DA precursor cells survived well in the striatum and SN and could further mature into DA neurons at both loci. Importantly, these DA neurons extended their neurites into the nearby space, which was similar to what was observed following transplantation of fetal ventral mesencephalon tissues into PD patients' striatum [20, 35]. GFP-positive DA neurons in the SN also extended neurites, and the direction of the neurites seemed to be random, not specifically pointing to the natural target area—striatum. The results suggest that the adult brain may lack molecular cues that could guide the axonal growth of the grafted DA cells at the SN towards in a direction towards the striatum.

iNSC-derived motor neuron precursors could also survive and mature into motor neurons following transplantation into a rat spinal cord, indicating a potential use in treating spinal cord diseases. However, survival seemed to be inferior with iNSC-MNPs engrafted in the rat spinal cord to the iNSC-DAPs in the monkey striatum. The exact reasons are not clear. It could be due to the intrinsic difference between iNSC-MNPs and iNSC-DAPs; it may also be attributed to the different extent of immune responses in brain vs. spinal cord or in monkey vs. rats. Future studies using strict parallel controls are needed to address this issue.

## 5. Conclusions

iNSCs reprogrammed from human PBMNCs could be differentiated into different neural precursors, which were able to survive and mature following transplantation into various areas of the central nervous system of a nonhuman primate and rats. iNSCs may offer a potential cellular source for treating neurological diseases.

## Data Availability

The data used to support the findings of this study are available from the corresponding authors upon request.

## Supplementary Materials

Supplementary Materials Supplementary Figure 1: knock-in of GFP gene at AAVS1 locus. Supplementary Figure 2: safety and specific differentiation of iNSCs transplanted at the motor cortex and of iNSC-DAPs at the striatum in the brain of a nonhuman primate. Table S1: primers used in qRT-PCR experiments. Table S2: all antibodies used in immunofluorescence staining experiments.
