# Supplementary material for: Characterization of Human-Induced Neural Stem Cells and Derivatives following Transplantation into the Central Nervous System of a Nonhuman Primate and Rats
Source: Stem Cells Int. 2022 Dec 28;2022:1396735. doi: 10.1155/2022/1396735 (PMC9812602; doi:10.1155/2022/1396735)
Supplement: Supplementary Materials — Supplementary Figure 1: knock-in of GFP gene at AAVS1 locus. Supplementary Figure 2: safety and specific differentiation of iNSCs transplanted at the motor cortex and of iNSC-DAPs at the striatum in the brain of a nonhuman primate. Table S1: primers used in qRT-PCR experiments. Table S2: all antibodies used in immunofluorescence staining experiments. [file 1396735.f1.pdf]

Supplementary Information

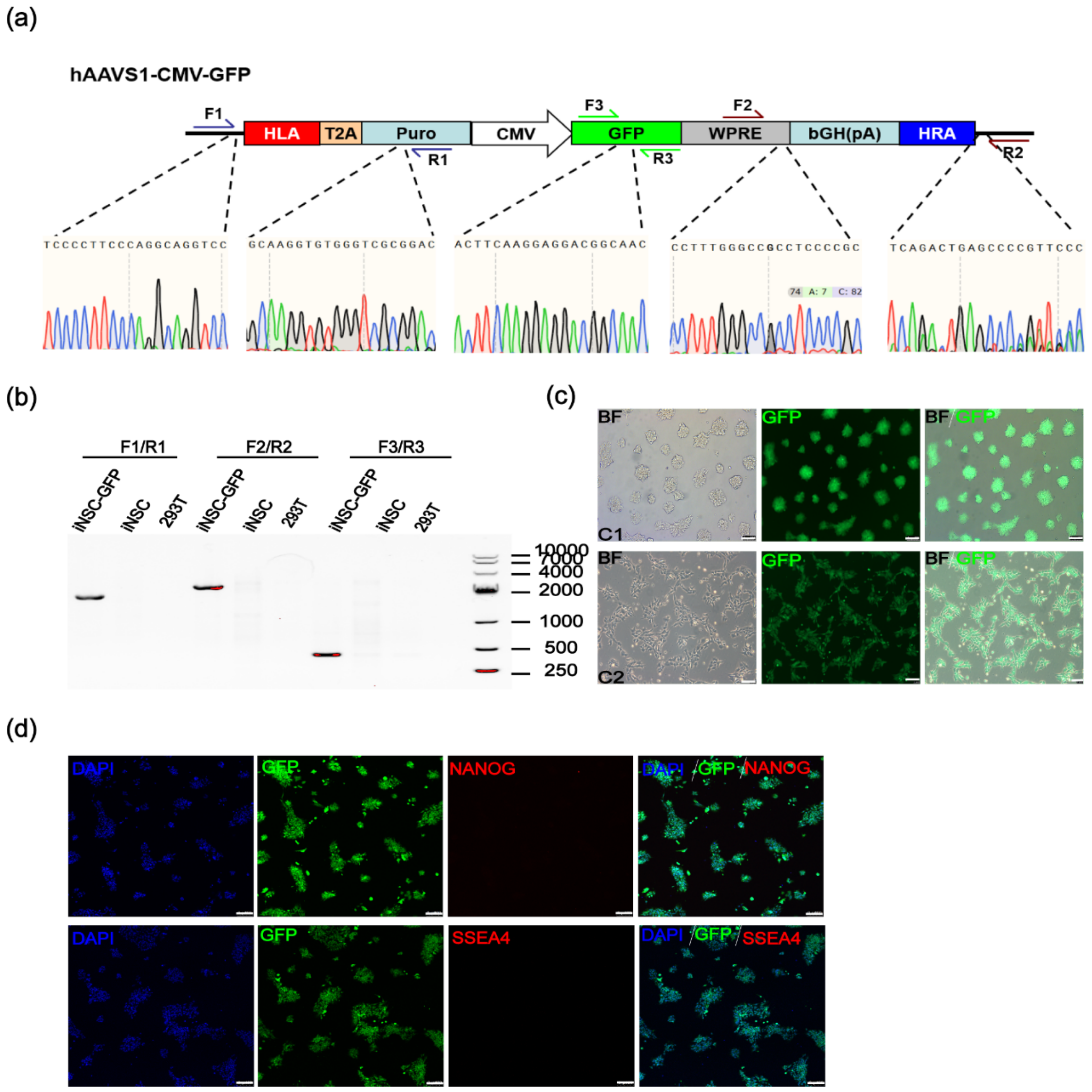

Figure S1. Knock-in of GFP gene at AAVS1 locus.

(A) Schematic diagram of site-specific knock-in of GFP gene at AAVS1 locus and DNA sequencing results using F1/R1, F2/R2 and F3/R3 primers. (B) Validation of knock-in of GFP using three pairs of primers on the cells of iNSCs-GFP, iNSCs, and 293T. (C) GFP expression in iNSCs cultured in proliferation (C1) and differentiation (C2) medium, respectively. Scale bars, 100 μm. (D) iNSCs-GFP stained negative for pluripotent markers SSEA4 and NANOG. Scale bars, 100 μm.

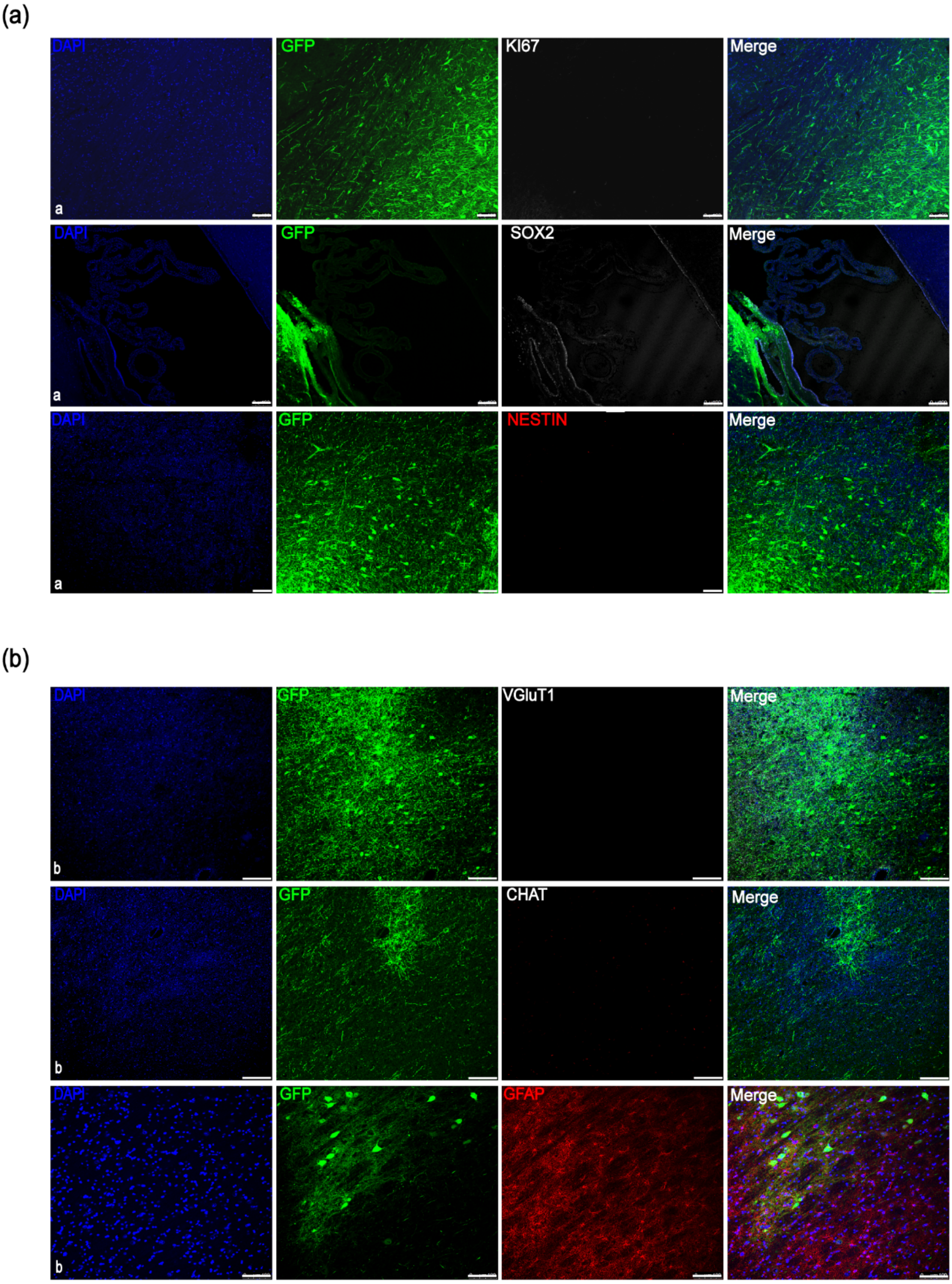

Figure S2. Safety and specific differentiation of iNSCs transplanted at motor cortex and of iNSC-DAPs engrafted at striatum in the brain of a nonhuman primate.

(A) iNSCs transplanted at the motor cortex were negative for KI67, SOX2, or NESTIN three months following engraftment. Scale bars, 100 µm. (B) iNSC-DAPs transplanted

at the striatum stained negative for glutaminergic neuron marker, VGluT1, cholinergic neuron marker, CHAT, and astrocyte marker, GFAP three months following engraftment. Scale bars, 100 μm.

Table S1. Primers and product length of PCR for each gene.

Gene	Forward (5'-3')	Reverse (5'-3')	Product length (bp)
SOX2	AGTCTCCAAGCGACGAAAAA	TTTCACGTTTGCAACTGTCC	189
FABP7	TGTGACCAAACCAACGGTAAT	CTTTGCCATCCCATTTCTGTA	200
HES5	GCGACCGCATCAACAGCA	GCGTGGAGCGTCAGGAACT	235
PAX6	GGTGAGAAGTGTGGGAACCG	GTGCTGCTGTTGTTGCTTGA	183
SOX1	GTTTTTTGTAGTTGTTACCGC	GCATTTACAAGAAATAATAC	173
FOXA2	GGAGCAGCTACTATGCAGAGC	CGTGTTCATGCCGTTCATCC	83
LMX1A	ACGTCCGAGAACCATCTTGAC	CACCACCGTTTGTCTGAGC	248
TH	GGGCTGTGTAAGCAGAACG	AAGGCCCGAATCTCAGGCT	107
NURR1	ACCACTCTTCGGGAGAATACA	GGCATTTGGTACAAGCAAGGT	175
β-Actin	CTTAGTTGCGTTACACCCTTTC	ACCTTCACCGTTCCAGTTTT	145
F1/R2	GCAGTCCTCCTTACCATCC	CCAGAATAGAATGACACCTAC	2022
F2/R2	TCAATCCAGCGGACCTTC	CAGAGACAGTGACCAACCA	2636
F3/R3	GCAGCACGACTTCTTCAA	GAACTCCAGCAGGACCAT	433

Table S2. Antibody information.

Primary antibody	Dilution	Vendor	Host
TH	1:500	Abcam	Rabbit
NURR1	1:500	Abcam	Rabbit
GIRK2	1:500	Abcam	Goat
CHAT	1:500	Millipore	Goat
FOXA2	1:500	Abcam	Rabbit
MNX1	1:500	Abcam	Rabbit
KI67	1:500	Proteintech	Rabbit
GFAP	1:500	Biolegend	Mouse
SOX2	1:500	Abcam	Mouse
ZO-1	1:500	Abcam	Rabbit
NESTIN	1:500	Abcam	Mouse
PAX6	1:500	Abcam	Rabbit
OLIG2	1:500	Abcam	Rabbit
N-CADHERIN	1:500	Abcam	Rabbit
NANOG	1:500	R&D System	Goat
SSEA4	1:500	Abcam	Mouse
VGluT1	1:500	Abcam	Rabbit
